# Fair food futures UK: Protocol for a mixed methods study exploring what approaches adopted by community food organisations are more likely to prevent the need for emergency food in two multicultural communities in Northern and Southern England

**DOI:** 10.1371/journal.pone.0304917

**Published:** 2025-02-10

**Authors:** Laura Sheard, Giorgia Previdoli, Wendy Burton, Rachel Benchekroun, Maddy Power, Bob Doherty, Philip Hadley, Ariadne Beatrice Kapetenaki, Shahid Islam, Sairah Mirza, Claire Cameron, Kate Pickett, Angela Hutton, Charlotte Edwards, Maria Bryant

**Affiliations:** 1 Department of Health Sciences, University of York, York, United Kingdom; 2 Thomas Coram Research Unit, University College of London, London, United Kingdom; 3 School for Business and Society, University of York, York, United Kingdom; 4 Bradford Institute for Health Research, Bradford, United Kingdom; 5 City of Bradford Metropolitan District, Bradford, United Kingdom; 6 London Borough of Tower Hamlets, London, United Kingdom; 7 The Hull York Medical School, York, United Kingdom; PLOS: Public Library of Science, UNITED KINGDOM OF GREAT BRITAIN AND NORTHERN IRELAND

## Abstract

**Introduction:**

Food insecurity reduces people’s chances to live healthy and active lives and places a significant burden on healthcare systems. Levels have significantly increased in the UK since 2010, due to the impact of austerity and, more recently, the COVID-19 pandemic and the cost of living crisis. This increase is projected to continue. Households with children are amongst those at highest risk for food insecurity. A variety of community food organisations (CFOs), such as community gardens, community kitchens, food banks and social markets, have been essential in responding to rising food insecurity, including providing emergency food and other types of support such as welfare advice. However, beyond food banks, little is known about differing approaches to food aid in the UK, including how these organisations provide additional services to address the underlying issue that has led someone to seek emergency food support.

**Aim:**

To understand what approaches used by community food organisations are most likely to help prevent the need for emergency food in two multicultural communities in the North (Bradford) and South (Tower Hamlets, London) of England, with high levels of ill-health and food insecurity.

**Research design, setting and participants:**

This is a mixed methods study informed by complex systems theory. Methods include participatory systems mapping and qualitative longitudinal research. We will map the availability and type of help with food, and produce a typology of CFO approaches, using a survey, multiple local and national participatory system mapping workshops and interviews with local and national stakeholders (WP1). Then, we will conduct a longitudinal qualitative research using a ‘researcher in residence’ approach in up to 10 CFOs purposively sampled to reflect the diversity of prevention strategies adopted by CFOs. Research will include: a) a 12 month ethnographic study; b) three waves of ‘go along’ interviews with up to 35 families; and c) a visual study where the same families are invited to share photos and videos about their food thoughts via Indeemo research app.

**Outputs and dissemination:**

Outputs will include: a) a toolkit on CFOs to support local and national policy and implementation decisions, b) a travelling exhibition with visual representations of people’s lived experiences c) publications in academic journals, d) blog posts, e) public talks, and f) policy briefs. Findings will help decision makers to invest in the most accessible, beneficial and culturally appropriate resources for communities.

## 1. Introduction

### 1.1 Food insecurity and health inequalities

#### Food insecurity and health

International research has shown that food insecurity is a cause and consequence of poor health [1–4] and places a significant burden on healthcare services [1, 5]. People are described as food insecure ‘when they lack regular access to enough safe and nutritious food for normal growth and development and an active and healthy life’ [6].

Thus, while ‘food insecurity’ is often used interchangeably with food poverty [7], it refers both to the availability of safe and nutritious food to conduct a healthy life, and to concerns around the ability to access food in the future [6, 8]. Some definitions include aspects beyond nutrition, such as social acceptability, taking into consideration wider social and cultural components embedded in the experience of food [9, 10]. Some also consider food insecurity as a manifestation of poverty, linked to wider social issues, rather than a separate matter and contest its ‘medicalisation” [10, 11]. In the UK, food security only began to be routinely monitored in 2019, so evidence of its impact on health outcomes is less extensive. Data so far indicate that diet-related ill health causes preventable illness and contributes to health inequalities [12] and that food insecurity can affect people’s mental health [13, 14].

#### Policy interest in food insecurity and initiatives by campaigners

There is ongoing and high-profile policy interest in the impact of food insecurity on well-being [15]. In 2022, the NHS Confederation made a call to national and local governments to address food insecurity to prevent devastating and long-lasting impacts on people’s health and wellbeing [5] and Public Health England recommended a strategic and comprehensive response to food poverty [16]. The Department for Levelling Up, Housing and Communities recognised the need for affordable, accessible and healthy diets in their White Paper [17] and the Department for Environment, Food & Rural Affairs highlighted the contribution played by local food partnerships, bringing together councils, community groups and businesses in “addressing food affordability and accessibility to healthy food” in their Food Strategy White Paper [18]. In May 2023, the Scottish Government published a human rights inspired three year plan to end the need for food banks in Scotland [19]. All-Party Parliamentary Groups have produced evidence based recommendations [20]. At the same time, grassroots organisations and charities, including the two main food bank networks (Trussell Trust and Independent Food Aid Network), are calling for changes to the welfare system and campaigning for political action to end the need for food banks across the UK [21].

### 1.2 The scale of the problem in the UK

In the UK, families with young children are particularly at risk of experiencing food insecurity [22–24]. COVID-19 has precipitated a significant increase in food insecurity [13, 25] made worse by the rapidly growing cost of living [26].

In recent years, different organisations started collecting data on the prevalence of food insecurity in the UK. Data are collected by the Department for Work and Pensions (DWP) as part of the Families Resources Survey [27] and by the Food Standards Agency, as part of the Food and You 2 Survey [28]. The Food Foundation is assessing the impact of household food insecurity across the UK through periodical surveys (Food Insecurity Tracking [29]). Differences in methods, frequency of data collection and population are reflected in the results: In 2023, the percentage of UK households who reported having experienced moderate or severe insecurity were 10% according to DWP [30] and 17% according to the Food Foundation [31].

Data show that households with members receiving Universal Credit were four times more likely to have experienced food insecurity (41% vs 10% according to DWP [30]). Compared with households without children, households with children were at higher risk of experiencing food insecurity (23.4% vs 14.8%) [31] and, if children were aged 6 or younger, the risk increased significantly [32]. Households from ethnic minority groups were more likely to experience food insecurity, compared to households from a White British background [31]. The Food Insecurity Tracking [29] shows an overall decrease in the percentage of households reporting food insecurity (from 17% in June 2023 to 14.8% in January 2024), but families with children still appear to be worse affected. In 2024, one out of five households with children reported experience of food insecurity (20%) [33], with the proportion going to almost one in four (23.6%) in households with one or more children under 4 [33].

Research has drawn attention to the relationship between austerity policies and food insecurity in the UK [34–36], including the role that welfare reform has played to increase food bank usage [37, 38].

### 1.3 Roles played by community food organisations

Community food organisations (CFOs) are local food infrastructures that can respond to community food insecurity and can be locally or nationally managed [39]. They are varied in their approach and include provision of ‘emergency food’, such as a selection of ‘essential’ non-perishable food items. CFOs include community gardens, food pantries, community supermarkets, urban farms, community kitchens, food banks and other community and grassroots organisations campaigning to end food poverty [40–44]. There has been rapid growth of CFOs over recent years [45] and particularly since the COVID-19 pandemic [46, 47]. Both independent [48] and Trussell Trust affiliated food banks [49] reported a surge in demand in 2022–2023, especially from people accessing food aid for the first time, despite the fact that many people in need have previously not used CFOs [7, 50], perhaps due to stigma and shame [36, 51]. International evidence suggests that CFOs can contribute to alleviating food insecurity, by ‘providing immediate solutions to severe food deprivation” [52]. Researchers, though, have expressed concerns that emergency welfare provision, such as emergency food, is increasingly getting incorporated in the main UK welfare system [53]; while national campaigns have been calling for responses addressing underpinning issues, like benefits unfitted to match increased cost of living [21].

Local responses to food insecurity vary in many aspects [54]. Formal food banks, which operate via recipients receiving referrals from professionals (e.g. Trussell Trust), have been widely studied [55, 56]. There is little research, though, to support understanding of what approaches, models and components adopted by CFOs are most beneficial at reducing the need for emergency food, and the associated impacts among diverse groups, in both the short- and long-term, are not known [57]. “Models” here indicate the mode of delivery of food aid (e.g. drop in, home delivery or referral), while ‘components’ refer to activities and actions (e.g. welfare advice, employment support, food vouchers, emergency provision of food). Public health classification of prevention strategies (primary, secondary, tertiary) [58] can help us to understand how CFOs respond to food insecurity. A primary prevention informed approach, in this perspective, includes all initiatives to improve financial security, like for example enhancing educational or employment opportunities to prevent people from experiencing food insecurity. A secondary prevention approach includes the offer of advice, such as welfare rights advice, to prevent insecurity from worsening. And, finally, tertiary prevention refers to the attempt to mitigate the immediate impact of food insecurity via provision of food [59].

Recent research looking at the effectiveness of food banks at reducing food insecurity has focused on assessing the nutritional quality of the food provided [60]. But research on inclusivity, access and impact of the different types of CFOs is still limited. There is a need for research to generate rich evidence to inform culturally appropriate, sustainable, community-based policies which can be applied to other (ethnically diverse) populations.

Local authorities linked to the research team and the Department of Food, Environment and Rural Affairs (DEFRA) have expressed that there is a need to better understand the preventative capabilities of CFOs to better target resources so that communities can flourish. Efficient and effective targeting of resources is crucial given the impact that the ongoing increase in cost of living is having on lower income households [61, 62]. Thus, the research described here will explore the impact of CFO approaches, models and components (what works, for whom, under what circumstances) by considering the impact of CFOs among multi-ethnic populations in the North and South of England.

## 2. Materials and methods

### 2.1 Research aim and objectives

This research aims to investigate what approaches used by community food organisations are most likely to help prevent the need for emergency food in two multicultural communities in the North and South of England. This research will inform local and national governments as to how best to invest in these or other resources. Using a flexible and adaptive approach, our primary objectives are:

1. To conduct stakeholder workshops and interviews to position the role of CFOs amongst other factors that influence food insecurity, including the types of approaches used to prevent the need for emergency food aid; exploring inter-dependencies in the system and generating a typology of approaches (work package 1).
2. To undertake a longitudinal qualitative study to understand whether, how and in what ways different CFO models and components (identified in Objective 1) provide primary, secondary or tertiary prevention of food insecurity and the impact these have on the food security status of families (work package 2).
3. To ensure that our findings have the greatest benefit so that (a) the most effective prevention models and components can be adopted by CFOs, (b) food vulnerable communities can access the most beneficial and culturally appropriate resources and (c) local and national governments can best target available funds to reduce the need for emergency food (work package 3).

### 2.2 Research design

**Fig 1:**
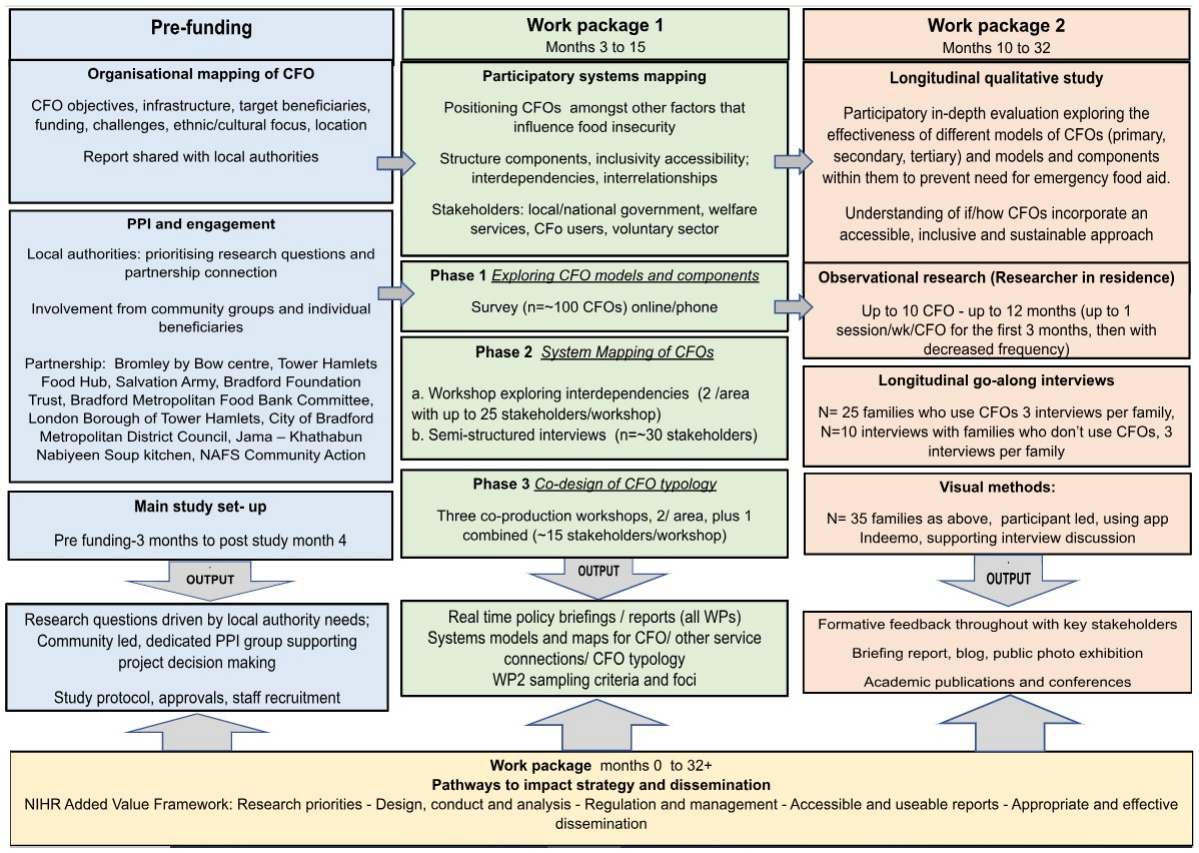
Design and work packages.

This flexible protocol describes a research study with three integrated work packages involving: systems mapping (WP1), a longitudinal qualitative study (WP2), and a work package specifically dedicated to impact and implementation (WP3) (Fig 1).

**Fig 2:**
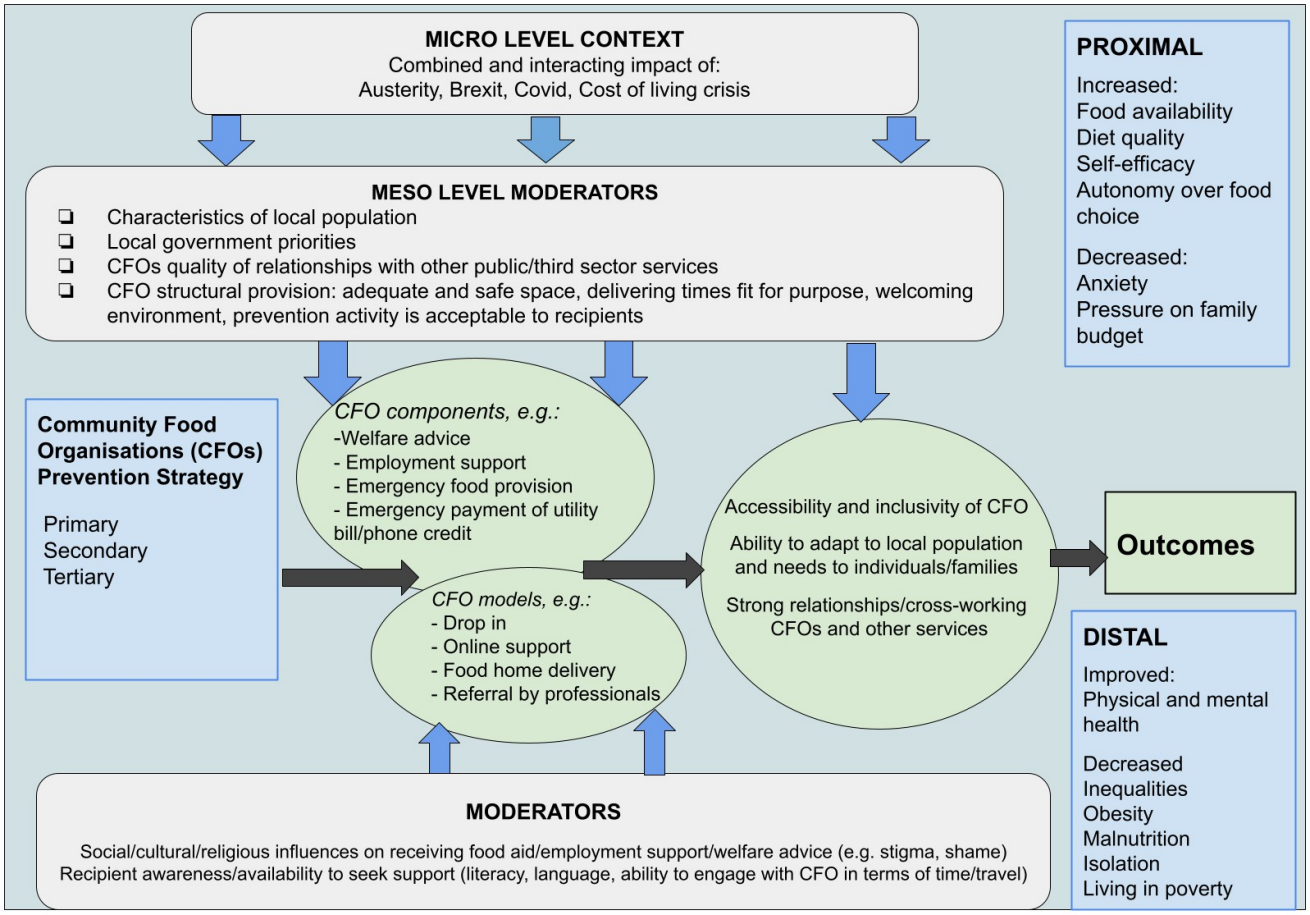
Logic model.

Methods and activities are all underpinned by a complex system thinking approach [62]. The research team will evaluate the inherent complexities of between five and ten CFOs across Bradford and London, within the wider food and welfare systems in which they sit, whilst paying attention to the specific populations and communities they serve and the factors that prevent dependence on emergency food aid [63–67]. A dynamic logic model [68] which highlights our assumptions about how CFOs produce their outcomes, allowing for dynamic and adaptive directions to emerge and/or flex has been developed to support this evaluation (Fig 2).

### 2.3 Research setting

Our research settings are in Bradford and Tower Hamlets, London. Both areas are ethnically diverse with high levels of poverty and ill health [69, 70]. 29% of children (under 16) living in Bradford [71] and 22% in Tower Hamlets [72] live in absolute poverty. When taking into account housing costs, the estimated percentage of children living in low income households in Tower Hamlets increases to 56% [73] and 39% in Bradford [74]. Pre-COVID, 14% of Born in Bradford cohort families [75] were food insecure [76] but this increased to up to 20% after the start of the pandemic [77]. At about the same time, only half of the families who responded to a survey in Tower Hamlets reported an ability to afford a balanced meal [78].

Previous work conducted with families identified ethnic differences in the complexity of the issue and suggested a potential protective effect of ethnic minority social networks for some groups [10, 13, 76, 79] and found that some groups are less likely to use CFOs even at times of need. Previous exploratory work in Bradford described the location and objectives of a large, varied and complex network of different types of CFOs across Bradford [36]. At that time, relatively few CFOs operated as food banks [80], perhaps reflecting adaptation to the cultural and religious needs of the population. Comparable work was done in Tower Hamlets, suggesting similar complexity [81].

### 2.4 Work Package 1 (WP1): Systems mapping (Months 3–15)

#### 2.4.1 WP1 Research questions, phases and theoretical underpinning

WP1 has three research questions:

1. What are the different organisational models and components of CFOs?
2. What do the social and economic systems in which CFOs operate look like?
3. What are the key organisational components and political pathways for successful/unsuccessful implementation of services provided to achieve inclusivity and accessibility, and to reduce the need for emergency food?

Adopting a systems approach within WP1 will allow us to: 1) explore interactions and interdependencies between the CFOs; 2) identify complex drivers of the use of food support (e.g. socio-economic/health factors); and 3) investigate outcomes of CFOs (e.g. individual and community level food security).

#### 2.4.2 WP1 methods

WP1 Phase 1: Exploring CFOs Models and Components

Sampling and recruitment: Informed by piloted methods [80], Phase 1 will provide a detailed descriptive picture of CFOs in the two locations, Bradford and Tower Hamlets via combining publicly available information (e.g. information openly provided on organisational websites) with survey data completed by consenting CFO representatives.

Individuals from CFOs who agree to complete the survey will be asked to recommend other CFOs to approach (snowballing approach). This will be done until data saturation is reached (based on our previous similar work, this will include survey completion by approximately 100 organisations).

Data collection: structured online survey questionnaire will seek information on:

- nature and frequency of service provision
- demographics of service users
- accessibility and cultural specificity of the service
- connections with other service providers.

WP1 Phase 2: Systems mapping of CFOs

Sampling and recruitment: Phase 2 will apply a systems approach in a participatory way, capturing multiple perspectives of the food insecurity welfare system. Two workshops per location (up to 25 stakeholders per workshop) will be conducted; the first with local stakeholders and the second with CFO users and people with lived experience of food insecurity. Building on relationships established in Phase 1, stakeholders and CFO users will be invited to the workshops via sampling from a range of sectors. We will then conduct semi-structured interviews with representatives of food aid providers at local and national levels (N = 30) to probe the key themes mapped in the workshops and further explore interdependencies between different services (going beyond the immediate CFO system). Interviewees will be recruited from national and local voluntary organisations; welfare services; national and local government; the food sector; and other delivery organisations, including those who take part in our workshops. We will apply a sampling framework to ensure representation (i.e. role/sector, location, approach). In addition to invitations using existing networks (and those identified during Phase 1), participants will be signposted by our partners (e.g. DEFRA, local authorities).

Data collection:

Each workshop (~3 hours) will involve an exploratory (first part) and confirmatory (second part) group model building process [82]. In the first part, participants will be asked to identify factors involved in (and influencing) the CFO system and describe how these relate. The second part will review both the findings of the exploratory component and the Phase 1 findings. A draft map will be built during the session, which will be reviewed and finalised in Phase 3.

Semi-structured interviews will be conducted face-to-face or via phone/Zoom, as appropriate. Interviews will last approximately one hour, conducted at a time and place convenient for the interviewee, recorded and transcribed verbatim.

All participants will be provided with full information about the study before agreeing to take part, including a written information sheet.

WP1 Phase 3: Co-design of CFO typology

Sampling and recruitment: Three participatory workshops with local and national stakeholders will be conducted to co-design a systems map and typology of CFO models and the socio-political systems in which they operate. Each workshop will include up to 15 stakeholders.

Data collection: One workshop in each location will ask participants to consider findings of earlier work to review and agree on draft CFA typologies and systems maps. A final online national workshop (2 hours) will then review and agree on a theoretical typology, based on the findings of prior workshops and data collection.

#### 2.4.3 WP1 analysis

Survey and desk research data will be analysed descriptively, including basic quantitative representations of models and components. System mapping workshops will produce a systems maps [83]. We will explore leverage points applying the Action Scales Model, [84]. All qualitative data (e.g. systems mapping workshops and interview transcripts) will be analysed thematically.

### 2.5 Work Package 2 (WP2): Longitudinal qualitative study (Month 10–32)

#### 2.5.1 Research questions and theoretical underpinning

*Primary research question*:

1. Which CFO models or components are most beneficial at reducing families’ need for emergency food? How and in what ways?

*Secondary research questions*:

2. How do different CFO models and components interact with the wider system, and to what degree is this interaction beneficial to food support recipients, local authorities and food organisations?
3. When explored longitudinally, what is the role that different CFO models and components play when thinking about people’s mental and physical health and their own sense of food security?
4. Does the way that differing models and components of CFO interact with, and benefit, families differ across different population groups (level and longevity of food insecurity; family composition, ethnicity and religion)?
5. Do specific CFO models and components i) confer positive or negative unintended consequences ii) introduce, reinforce or weaken health inequalities?

Theoretical underpinning

Following Hawe [63], in this study, research sites/ communities will be viewed as complex ecological systems which are contingent on setting, social networks and temporality. This research will examine the impact that a CFO has on the system within which it operates and on the lives of the individuals with which it interacts. The theoretical approach adopted is critical to understand whether, how and why different CFOs are able to bring about sustainable change and more specifically which CFO models, components and prevention approaches are able to reduce the ongoing need for emergency food.

#### 2.5.2 Sampling and recruitment of CFOs

Between two and five CFOs in Bradford and Tower Hamlets (up to 10 in total) will be identified via the systems mapping and typology of CFO components conducted in WP1. A diverse range of CFOs will be intentionally included, such as under-researched forms of CFOs, for example those which are responsive to local populations in terms of religion or ethnicity or have a more informal structure. The full range of CFOs is not yet known but could include: religion-based food provision (a church or a mosque having a stall with free food or organising free social meals for their communities), community supermarkets run by schools staff or parents or churches, community cafes, provision of food parcels or meals on the street by informal groups of volunteers, community gardens or farms, pantries offering reduced price food to members, and more or less formalised food banks. Any of these CFOs could deliver aspects of primary and secondary prevention such as co-located welfare or employment support. Individual CFOs could include components of one, two or all three approaches (primary, secondary and tertiary), reflecting real work complexity. This purposive sampling approach will also ensure that examples of primary, secondary and tertiary prevention approaches [85] are all included.

We will shortlist around 30 CFOs, to allow maximum variation around the criteria that the systems mapping will identify as most meaningful (e.g. size, referral path, type of organisation, how help with food is delivered, what additional services are on offer, prevention level.). We will then arrange short, informal visits to the shortlisted CFOs to further explore how models, components and prevention approaches described by staff in the survey, workshops or in online material, translate into real world practice. We will not collect data during these visits. Researchers’ informal observations from the visits and follow up discussion with the wider team will inform the final decision about which CFOs should be formally invited to take part in the study.

#### 2.5.3 WP2 methods

**Fig 3:**
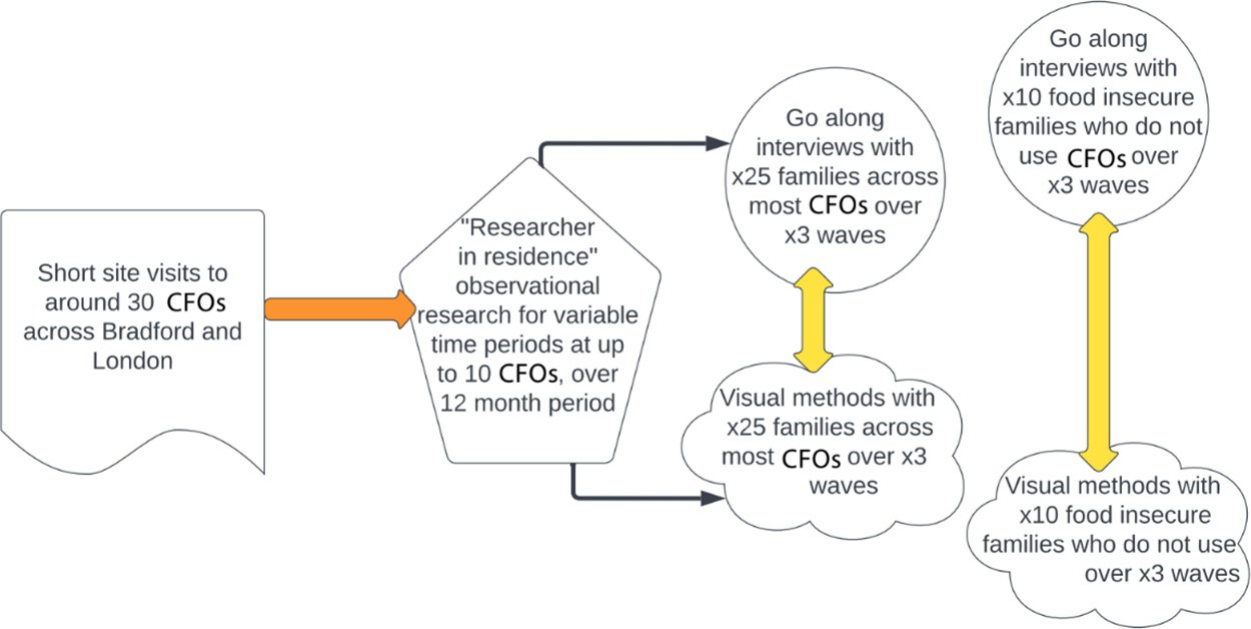
Methods in work package 2.

WP2 will use three longitudinal qualitative methods: 1) Observational research via a Researcher in Residence model 2) Go along interviews with families using CFOs and families who are not 3) Visual study with both the above family groups (Fig 3).

WP2 Method 1: Observational research via Researchers in Residence

Approach and tools: This is a 12 month fieldwork period during which study researchers will take on the role of ‘researchers in residence’ and become CFO volunteers, in order to embed themselves in the daily life of each CFO. First used in healthcare research, the Researcher in Residence (RR) approach [86] encourages real relationship formation, trust and rapport to develop between the researcher and those around them. In the RR model, the key relationships are between research, policy and practice. This implies that the learning from the research can feed straight into local policy decisions and immediate CFO practice, rather than primarily addressing an academic audience. It potentially also increases reciprocity at organisational level, for example by offering CFO leads meaningful learning in exchange for hosting a researcher.

Data collection: Over a 12 month period, the RRs will spend time grounding themselves in the CFO, observing everyday life and talking to people informally [86]. RRs will be experienced qualitative researchers with a background in ethnographic research and who have previously worked with marginalised people. There will be 2 RRs in Bradford and 1 RR in London.

Each RR will spend up to one session per week at each CFO in which they are hosted for approximately three months. As familiarity grows, the time spent in each CFO is likely to reduce down to around a session every two/three weeks. During the first three months, the researchers will naturally meet people using the CFO and begin to build rapport with people to undertake ‘go along’ interviewing. The opinions and experiences of staff and volunteers will be gathered through informal conversations that opportunistically arise over approximately 12 months.

Observational foci:

The RRs will take on volunteer work at each of the CFOs to embed themselves (the extent and nature of this will be in agreement with each CFO). RR tasks could include receiving deliveries, sorting food, making food parcels and distributing food. RRs may also be trained to support people to claim benefits or seek employment or signposting to other services. The role of RRs will be intrinsically relational: it will involve listening to people who may be in crisis, understanding people’s frustrations and fostering a warm and supportive environment, often involving a cup of tea. Undertaking all of the above does not mean that the RR therefore becomes ‘biased,’ as a key requirement of the role is continual reflection of independence.

Consent procedure:

The RR will meet with CFO staff and volunteers prior to fieldwork commencing and written signed consent will be obtained from the CFO manager. People using the CFO will encounter the RR as a volunteer and, wherever possible, the RR will introduce into the conversation that they are a researcher, tell people about the study and gain verbal consent for their story to be included in field notes. This may not be appropriate for fleeting contact or when people are in distress. Extreme care will be taken to ensure that information reported in the field notes is never identifiable.

WP2 Method 2) Longitudinal go along interviewing Approach and tools:

Go along, participatory interviews will be conducted over three waves with 25 families who access CFOs and 10 families who are food insecure but do not access CFOs.

Go along interviewing is a person centred, participatory and interactive method that focuses on understanding changing experiences [87]. It has been used in a diverse range of topics such as exploring leisure experiences for people with mental health issues [88], older patients’ transitions from hospital to home [89] as well as exploring food practices and insecurity in later life [90].

WP2 “go along interviews” will involve a narrative interviewing style, following people’s everyday lives to understand their food insecurity status in relation to supportive strategies (including CFO models and components). The “go along” part relates to the researcher going (travelling) alongside the participant as they go about their everyday usual activities with researcher and participant talking along the way. This contrasts with a static one-off interview where both participant and researcher sit down in one place (or over video/phone).

Sampling of families who use CFOs:

Sampling of individuals at each CFO will be opportunistic and near universal. The RRs will have natural conversations with people they meet as they undertake the volunteer work assigned to them by the CFO. At the start, the RRs will aim for a maximum diversity approach. As time goes on, there will be a narrowing of focus (both purposive and theoretical), leading to a selection of participants who will be invited to take part in repeated go-along interviewing.

Around 25 families with children will be recruited across sampled CFOs in Bradford and in Tower Hamlets, London. Our definition of ‘family’ is a parent or several parents with at least one dependent child under the age of 18. ‘Dependent child’ can be in any sense: biological, step, adopted etc. A diverse spread of families in terms of ethnicity, religion, disability and family composition (e.g. single parents) will be purposively recruited. In multi parent households, only families where all parents are actively interested in taking part in the study will be included.

Sampling of families who do not use CFOs:

Six families in Bradford and four families in London who are food insecure but do not use CFOs will also be recruited. People in the research team’s existing networks (including community groups, schools and those who are working in the food system in both cities) will be asked to provide assistance in identifying these families. Additionally, participants who do use CFOs will be invited to invite, in case they have relatives, friends or neighbours who may fit these criteria (snowballing). Non CFO users are likely to be a convenience sample. The aim is not a direct comparison of these two groups (users and non-users) but rather to provide further contextual information as to why CFO models may not be suitable or accessible for everyone who experiences food insecurity.

Data collection:

Each family will be invited to take part in around three go along interviews lasting approximately 2 hours to half a day each. The three waves of go along interviewing will be evenly spaced out over a 12 month fieldwork period, where appropriate. Any variance in the number of go along days (minimum of two and maximum of four) will be driven by participant preference. Data may consist of between 38 and 75 ‘go along’ half days with a mixture of field notes and audio recordings, predominantly weighted towards field notes. The term ‘audio recordings’ here refers to a broad range of data which may naturally occur in the field. This may range from a few minutes’ duration about a matter that the participant deems significant for the researcher to record, through to a duration of 30–40 minutes where important context may be lost if an audio recording is not made (over and above field notes being taken).

Interviews will take place over the longitudinal period regardless of whether participants are still using the CFO they were originally recruited from (if appropriate). The aim is to understand broadly whether they have moved out of, are slipping in and out or have stayed living in food insecurity and the complex factors that may have enabled this. The go along interviewing method therefore allows us to understand how different parts of the system are bolstering participants’ food status (or not).

Interview foci:

It is difficult to specify precise topics in advance for go along interviewing as the researcher aims for a naturalistic conversation [88] based on the activities each participant undertakes that day. Conversations may focus on: health, food, relationships, culture, ethnicity/race, discrimination and racism, religion/faith, shopping, housing, feelings associated with needing help with food insecurity, isolation, the benefits system, inequalities. The researcher will frame some questions on macro, meso and micro level moderators in our dynamic logic model and from key domains arising in our WP1 systems map. The conversation will be participant-driven.

Consent procedure:

Written, informed consent will be received from one person in the family unit, but the researchers will check verbally with other people who are likely to be observed that they explicitly consent. Researchers will also clarify that consent is not universal and that participants can ask the researcher at any time to not write field notes about a particular situation/event. If children are present, parental permission and children’s assent will be sought as appropriate.

WP2 Method 3) Visual /scrapbook study

Approach and tools: A predominantly visually-driven sub-study using a mobile phone app (Indeemo) where data (photos, videos, audio, other media) is collected by participants themselves over three waves.

Visual methods have been used previously in qualitative research projects focusing on food insecurity such as: older people’s susceptibility to malnutrition in the UK [90]; the experience of food insecurity among single men in Scotland [91] and the experience of food insecurity among parents who were veterans in the USA [92]. Photo elicitation is a technique whereby study participants are asked to take photographs of things that are meaningful to them regarding the research topic and the photograph is then used as a catalyst for discussion in a further research encounter, such as a go along interview. Photographs simultaneously incorporate a sensory element better suited to food-based research than a one-off interview where participants may struggle to articulate everyday taken-for-granted assumptions regarding food [93].

Sampling:

Participants who are involved in the go along interviews will be invited to take part in the visual study. Considering that a small number of families may not want to submit photos/videos due to cultural or privacy reasons, the sample for this visual methods stage is set up around 20 CFO using families and eight non-CFO using families.

Data collection:

All go along interview participants will be invited to use a mobile phone ethnography app (Indeemo) to take self-selected photos and videos of things that are *meaningful to them* regarding food [94]. The purpose of this is twofold: 1) photos and/or videos may be used as a catalyst to engender discussion points during the go along interview akin to photo elicitation techniques and/or 2) they may stand alone as a visual depiction of what food and food insecurity mean to the participant. Indeemo allows text or audio to be added as a caption to photos or videos, creating a “scrapbook” effect. This scrapbook option will be demonstrated to participants but they will have the power to decide whether they want to use captions or not. Participants will use Indeemo over a one week period over three waves, loosely matched to go along interviewing timescales. Participants will receive a soft prompt through the app from the research team. The minimum photo/video submission will be one per go along interview episode (no maximum).

Ownership of a smartphone is high in the UK, including in low-income populations [95]; however, our preparatory work with CFOs indicates that some populations (particularly asylum seekers) may not have access. Smart phones, therefore, will be provided to those who do not already have their own and fund phone data to all participants. Participants will receive support to help them navigate the app, where appropriate. Indeemo is structured like a social media app, so it is likely that most participants will be familiar with its interface style.

Visual/media foci:

Participants may take photos of things such as: food they enjoy eating; food they eat on a day-to-day basis based on their circumstances; making a meal; buying or procuring the ingredients; children’s meals or children’s food preferences; a visit to the supermarket or local store or café/pub/restaurant; a visit to a CFO or interaction with other services; an abundantly or sparsely stocked fridge or cupboard; specific dietary requirements such as halal, gluten free or vegan/vegetarian. Participants may wish to also photograph non-food related phenomena which impact on their ability to buy or enjoy food.

Consent procedure:

The Indeemo app can embed a digital information sheet and consent form as the front page. One participant per family will be asked to provide written informed consent regarding the upload of their media within the app. Explicit consent for use of each individual photo or video selected for display in the travelling exhibition (see WP3) will be sought from participants.

#### 2.5.4 WP2 analysis

*Formative*. Regular feedback loops will embed into our analysis, with rapid findings predominantly derived from the Researcher in Residence observations. Headline findings will be fed back sensitively in an iterative loop cycle to local authorities, CFO staff/volunteers and people who use CFOs via briefing reports, presentations and targeted conversations.

*Summative*. Data collected across all three qualitative methods be brought together and analysed as a whole.

Descriptive analysis will generate a targeted understanding of a) what works, for whom, when and why, and b) which CFO models/components are most beneficial. Analysis will be structured using the pen portrait technique, which is specifically designed to integrate the data from multiple qualitative methods collected over time to answer applied research questions [96]. The fundamental principles of this technique are to: draw on all the methods used, narrate interactions and events of importance at key time points, describe change occurring over time, and provide a well-rounded and holistic account of the phenomena being studied. A crucial element is to notice what is happening between different waves of a longitudinal project and include explicit analytic commentary on this.

Theoretical analysis will reach high level conclusions by focusing our analytic attention on Hawe’s “theorising interventions as events in systems” [63], abductively coding our data against the ‘four courses of enquiry’ contained within this. Analysis will move backwards and forwards in iterative cycles between the theory and empirical data. This will involve understanding longitudinally: 1) how CFOs sit within their wider context; 2) track/map changes to key relationships over time; 3) understand how resources are distributed and/or transformed; 4) uncover displaced activities. ‘Sense checking’ of both the descriptive and theoretical analysis will come from PPI, CFO users and strategic stakeholders at early and interim stages. The emphasis will be on iterative development of three dynamic logic models (one per prevention strategy) to propose CFOs underlying logic.

### 2.6 Work Package 3 (WP3): Pathways to impact and dissemination

A dedicated “pathways to impact” work package has been planned to generate societal, academic and policy impact. It runs throughout the whole project and is guided by the NIHR Adding Value Framework [97]. Our strategy is to produce accessible and usable outputs which reach key stakeholders and wider society so that primary prevention can be strengthened and changes can be made towards food secure communities. An ‘Actionable Toolkit’ aimed at local authorities will be produced to provide information on the typology and glossary of different CFO components and guidance on what works for whom, when and where. Research partners and the Public Involvement group (see below) will advise on the format and approach of the toolkit, though it is likely to become a freely accessible web-resource. A Travelling Public Exhibition will be designed for display in both locations using photos and quotes taken from WP2. This will be a powerful platform to share research findings with the wider public, encourage a dialogue on the topic and to give voice to the most vulnerable within these communities.

### 2.7. Ethical aspects and Public Involvement

Ethical approval for this research project was obtained from the Research Governance Committee of the University of York Department of Health Sciences on 15th May 2023 ***HSRGC/2023/566/C*** and included WP1 (CFOs survey, workshops and systems mapping) and WP2 activities (ethnography, go along interviews and visual study).

Ethical aspects have been taken into great consideration in the design of this research study due to the sensitivity of the topic and the potential vulnerabilities of the population involved. Measures to minimise risks to participants and counterbalance—where possible—the imbalance in power between researchers and participants will include:

- recruitment of a Public Involvement Panel Group including people with lived experience of food insecurity to advise the research team on all aspects related to recruitment, wording of all public facing project’s documents (including participants information sheets), mitigating strategies to counterbalance imbalance of power
- providing all participants with full information in written in plain English or alternative language to enable them to give informed consent
- working with PPI members to produce clear and concise video animations to explain key element of the study and how people can take part
- ensuring that researchers in the field remind participants that participation is voluntary and withdrawal is possible at any time
- ensuring that transcriptions of observation notes and conversations are pseudonymised and any identifying details are changed or omitted to protect people confidentiality.
- Involving a scientific oversight committee, with expertise in the population, settings, topic and methods

## 3. Status and timeline of the study

The study started in March 2023 and is expected to be completed by October 2025.

A diverse Public Involvement Panel with 8 members was recruited in July 2023 to provide ongoing advice and support to the research team.

Recruitment of participants and data collection for WP1 started on 17 July 2023 and has now been completed for the following activities:

- WP1 Systems Mapping Online Survey
- WP1 Systems Mapping Workshops in Bradford and Tower Hamlets
- WP1 Typology Workshops
- WP1 Systems Mapping Interviews

All participants involved in the above activities gave their written consent to take part.

Sampling and recruitment of sites for WP2:

After shortlisting, 38 CFOs were visited by the field researchers to further explore models, components and prevention strategies adopted. Three organisations in Tower Hamlets and four organisations in Bradford have now been invited to take part in the ethnographic study. All of them have accepted and we have received written consent from their coordinators/managers.

We expect to start recruitment of families using and not using community food organisations (WP2 walk along interviews and visual study) from July 2024.

We plan to complete recruitment of participants for all work packages by October 2025.
